# Supplementary material for: Identification of photoperiod-induced specific miRNAs in the adrenal glands of Sunite sheep (Ovis aries)
Source: Front Vet Sci. 2022 Jul 22;9:888207. doi: 10.3389/fvets.2022.888207 (PMC9354845; doi:10.3389/fvets.2022.888207)
Supplement: Supplementary file 1 [file Data_Sheet_1.ZIP › Supplementary file/miRDeep2 file.zip!/miRDeep2 file/Know_Structure/SP42A2/oar-mir-376a.pdf]

miRBase precursor : oar-mir-376a
Total read count : 140
oar-miR-376a-5p read count : 95
oar-miR-376a-3p read count : 40
remaining reads : 5

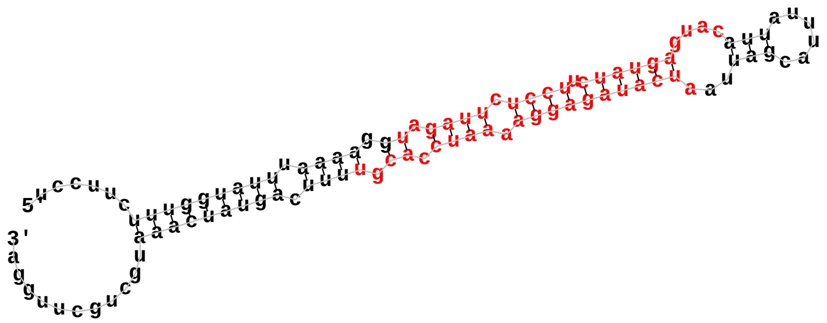

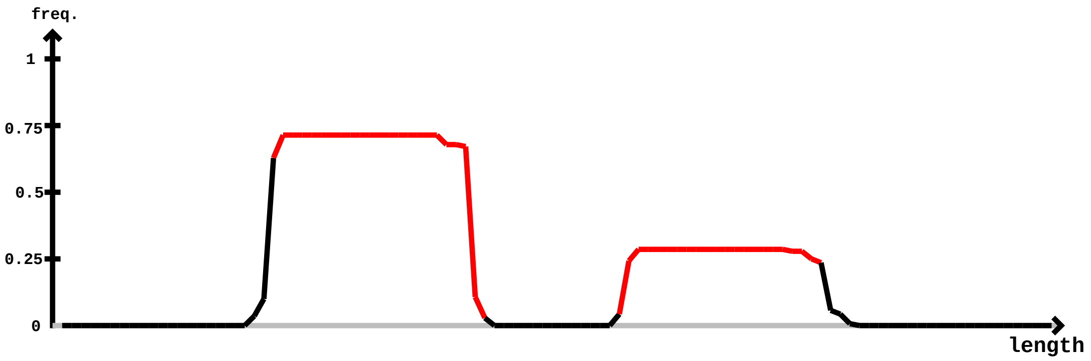


```
                                oar-miR-376a-5p
                                                                oar-miR-376a-3p
5'- uccuucuuugguauuuaaaagguagauucuccuucuaugaguacauuauuuacgauuaaucauagaggaaaauccacguuuucaguaucaaaugcugcuugga   -3'   exp
    ......((((((((.((((.((.((((.((((.((((((....(((......)))...)))))))))).)))).)).)))).)))))))))............   reads   mm   sample
    ...................agguagauucuccuucuaugagu.............................................................   5   0   seq
    ....................gguagauucuccuucuaugag..............................................................   1   0   seq
    ....................gguagauucuccuucuaugagu.............................................................   7   0   seq
    ....................gguagauucuccuucuaugagua............................................................   1   0   seq
    .....................guagauucuccuucuaug................................................................   3   0   seq
    .....................guagauucuccuucuaugagu.............................................................   58   0   seq
    .....................guagauucuccuucuaugagua............................................................   9   0   seq
    .....................guagauucuccuucuaugaguac...........................................................   4   0   seq
    ......................uagauucuccuucuaug................................................................   2   0   seq
    ......................uagauucuccuucuaugagu.............................................................   9   0   seq
    ......................uagauucuccuucuaugagua............................................................   1   0   seq
    .........................................................aaucauagaggaaaauccacg.........................   1   0   seq
    .........................................................aaucauagaggaaaauccacgu........................   5   0   seq
    ..........................................................aucauagaggaaaaucc............................   1   0   seq
    ..........................................................aucauagaggaaaauccac..........................   4   0   seq
    ..........................................................aucauagaggaaaauccacg.........................   1   0   seq
    ..........................................................aucauagaggaaaauccacgu........................   20   0   seq
    ..........................................................aucauagaggaaaauccacguu.......................   2   0   seq
    ...........................................................ucauagaggaaaauccacguuu......................   5   0   seq
    ...........................................................ucauagaggaaaauccacguuuu.....................   1   0   seq
```
